# The correlation between imaging expression of P16 and S100 in hypertrophic ligamentum flavum

**DOI:** 10.1186/s12891-020-03395-y

**Published:** 2020-06-08

**Authors:** Wei Hu, Yidong Liu, Shunli Kan, Tengfei Zhang, Zehua Jiang, Rusen Zhu

**Affiliations:** 1 Department of Spine Surgery, Tianjin Union Medical Center, Tianjin, 300121 China; 2 Department of Orthopaedics, Dagang Hospital of Tianjin Binhai New Area, Tianjin, 300270 China

**Keywords:** Ligamentum Flavum, Hypertrophy, P16, S100, Magnetic resonance imaging, Western blot, Quantitative PCR

## Abstract

**Background:**

Lumbar spinal stenosis (LSS) is a common degenerative disease, which can lead to neurological dysfunction and requires surgical treatment. In the previous study, we used H&E staining and immunohistochemistry to qualitatively analyze the expression of S100 and P16 in the pathological process of ligamentum flavum (LF) hypertrophy in patients with LSS. To further explore the relationship between P16, S100 and LF hypertrophy in patients with LSS, we quantitatively detected S100 and P16 and their expressed products based on molecular biology techniques, and analyzed their imaging correlation.

**Methods:**

Before posterior lumbar surgery, LF thickness was measured by Magnetic Resonance Imaging (MRI). Through the operation, we obtained the specimens of LF from 120 patients, all of whom were L4/5 LF. They were designated: simple lumbar disc herniation (LDH), single-segment spinal stenosis (SLSS), and double-segment LSS (DLSS). The detection of each side of LF was assessed. S100 and P16 and their expression products were detected by western blot and quantitative polymerase chain reaction (qPCR).

**Results:**

The dorsal mRNA expression of P16 in DLSS group was significantly higher than that in SLSS group. On the dorsal and dural side of LF, the expression of P16 mRNA and proteins in the LDH group was significantly lower than that in SLSS and DLSS groups. We found a correlation between the thickness of LF and the expression of P16. However, there was no significant difference in the expression of S100 mRNA and S100 protein on both sides of the ligament and among the three groups, and no significant correlation between the expression of S100 and the thickness of LF.

**Conclusions:**

P16 is involved in the process of LF hypertrophy in patients with LSS, and the imaging thickness of LF is related to the expression of P16. No obvious evidence proves that S100 may be related to the hypertrophy of LF in patients with LSS.

## Background

Lumbar spinal stenosis (LSS) is a common degenerative disease in modern life, and the hypertrophy of ligamentum flavum (LF) is an important factor leading to LSS [1, 2]. It is generally believed that the causes of LF hypertrophy include mechanical stress, inflammatory stimulation etc., and its specific mechanism has been the focus of international research. However, studies predominantly focus on the histological and imaging analysis of the cause of LF hypertrophy, and investigate its mechanism at the molecular level [3, 4].

Yoshida et al. studied the morphology and immunohistochemistry of LF, and ascertained that LF was mainly composed of elastic fibers and collagen fibers [5]. The pathogenesis of LF hypertrophy is predominantly proliferation, ossification and calcium crystallization deposition of type II collagen. LF is mainly composed of fibroblasts. Previous studies show that P16 is related to fibroblast senescence [6], and the inhibition of S100A8 protein may lead to the decrease of fibroblast growth and apoptosis [7]. We speculate that P16 and S100 may be related to the hypertrophy of LF. In our previous study, we used imaging and histological methods to grade the degree of LF elastin fibrosis, and an immunohistochemical method was used to detect the expression of P16 and S100 in ventral and dorsal LF. We found that the expression of P16 may be related to LF hypertrophy [8].

At present, there is no study to further compare the difference in expression between P16 and S100 at the molecular level of hypertrophic LF. The purpose of this study was to investigate whether the results of molecular biological expression were consistent with the results of previous histological and immunological studies, and to further explore the correlation between imaging findings and expression of hypertrophy of LF. We further aimed expound on the pathogenesis of LF hypertrophy and to provide a new way for the prevention and treatment of LSS.

## Methods

### Specimens collection

The research program was approved by the Institutional Review Committee of Tianjin Union Medical Center, and all procedures are based on the Helsinki Declaration. When patients underwent posterior lumbar surgery, the full thickness of the LF was removed from L4/5 segments. (*p* > 0.05, Table 3). There was no significant difference in baseline data between groups (*p* > 0.05, Table 1). After conservative treatment for at least three months, no obvious symptoms improved in all patients. All patients had no ossification of LF, secondary adhesive arachnoiditis, polyneuritis, no history of lumbar surgery, history of intraspinal invasive treatment such as epidural, etc. Considering the influence of diabetes and hypertension on the hypertrophy of the LF, none of the selected patients had a history of diabetes, hypertension, and underlying diseases that could have a potential impact on LF. We divided LF tissue into three groups: simple lumbar disc herniation (LDH), single-segment spinal stenosis (SLSS), double-segment spinal stenosis (DLSS). The dorsal and dural LF were detected for each group. Western blot and qPCR were used to detect S100 and P16 and their expression products.

**Table 1:** The baseline data of LDH, SLSS and DLSS groups

Group	Sample size	Sexual		Age	Course of disease
		Male	Female		
LDH	40	20	20	43.9 ± 13.2	83 ± 13.7
SLSS	40	19	21	45.5 ± 12.2	86 ± 11.4
DLSS	40	21	19	43.2 ± 11.3	92 ± 11.3
*F*		X^2^ = 0.934		F = 0.316	F = 0.689
*P*		> 0.05		> 0.05	> 0.05

### MRI measurement

The measurement method of LF was done as in our previous study [8]. We consider the ligamentum flavum> 3 mm as hypertrophy of ligamentum flavum in imaging measurement. The thickness of the LF was measured from the mid-point of the LF to the ventral side of the inner rim. The lumbar spinal canal oblique diameter is measured from the midpoint of the dorsal side of the ligamentum flavum to the midpoint of the posterior margin of the vertebral body. Relative thickness of LF (RT)(%) was calculated as the percentage of the thickness of LF to the oblique diameter of lumbar spinal canal. The average of three independent measurements of three surgeons were taken to determine the relative thickness of a single sample.

### Western blot

The dorsal and ventral LF of LDH, SLSS and DLSS were ground in liquid nitrogen. Extraction of total protein using RIPA lysate containing PMSF (Solarbio, USA). The CBA protein quantitative kit (Biosciences, USA) was diluted with 5 × buffer at 1:5 and denatured at 95 °C for 10 min To prepare SDS-PAGE gel (biorbyt, UK), 20 μL of total protein was taken, and the protein band was separated by 120 V electrophoresis for 1 h, then 100 mA transferred it to PVDF membrane (Millipore, USA). After 5% skimmed milk powder was sealed for 3 h, the PVDF membrane and the first antibody were incubated overnight at 4 °C. After washing the membrane 3 times with tris-buffered saline with Tween 20 (TBST, Solarbio, USA), the secondary antibodies labeled by Horseradish peroxidase (HRP, Solarbio, USA) were incubated at 37 °C for 1 h. The ChemiDocMP chemiluminescence imaging system (Bio-rad, USA) was exposed to observe the bands on the PVDF film.

### Quantitative polymerase chain reaction (qPCR)

Total RNA was extracted using Trizol reagent (Takara, Japan). SuperScriptIIIRT kit (Gibco, USA) is used for reverse transcription. Sybr qPCR mix (TOYOBO, Japan) was used for quantitative PCR analysis. PCR amplification conditions: 95 °C, 2 min, 94 °C, 20 s, 60 °C, 20 s, 72 °C, 30 s, a total of 40 cycles. Quantitative analysis was performed on ABI7900PCR instrument. Using Actin as the control, the primer sequences of quantitative mRNA, are provided in Table 2. The gene expression level was calculated using the 2^-ΔΔCt^ method relative to the actin gene. 2^-ΔΔCt^ > 2 or < 1/2 is considered statistically significant.

**Table 2:** P16, S100 primer sequence

Symbol	Forward primer 5′-3′	Reverse primer 5′-3′
actin	GACAGGATGCAGAAGGAGATTACT	TGATCCACATCTGCTGGAAGGT
P16	ATGGAGCCTTCGGCTGACT	GTAACTATTCGGTGCGTTGGG
S100	TGGCCCTCATCGACGTTTTC	ATGTTCAAAGAACTCGTGGCA

### Statistical analysis

The average values were taken from three biological repeats in each experiment. We determined the relationships between the thickness and the expression of P16 and S100 using Pearson’s correlation coefficient test. A one-way ANOVA was used to analyze the differences among the three groups. Use t-test for comparison between two groups. And all statistical analyses were performed by Statistical Program for Social Sciences (SPSS) software (version 21.0, IBM, New York, USA). All the analyses were statistically significant at *p* <  0.05.

## Results

### MRI measurement

The thickness of LF was measured and analyzed for a total of 120 times. Its absolute and relative thickness is shown in Table 3. Compared with SLSS and LDH group, the absolute and relative thickness of LF in DLSS group was larger (*p* <  0.05). The average thickness of DLSS group was 5.821 mm (rt = 44.501), while that of SLSS group and LDH group was 4.958 mm (rt = 37.420) and 2.811 mm (rt = 21.510), respectively.

**Table 3:** LDH, SLSS and DLSS absolute LF thickness, relative LF thickness

		LDH	SLSS	DLSS	*F*	*p*
LF thickness	Absolute (mm) ± SD	2.811 ± 0.612	4.958 ± 0.783	5.821 ± 0.731	74.160	< 0.01
	Relative (%) ± SD	21.510 ± 5.382	37.420 ± 8.591	44.501 ± 7.832	44.369	< 0.01

### The protein expression of P16 and S100 in each group

**Fig. 1:**
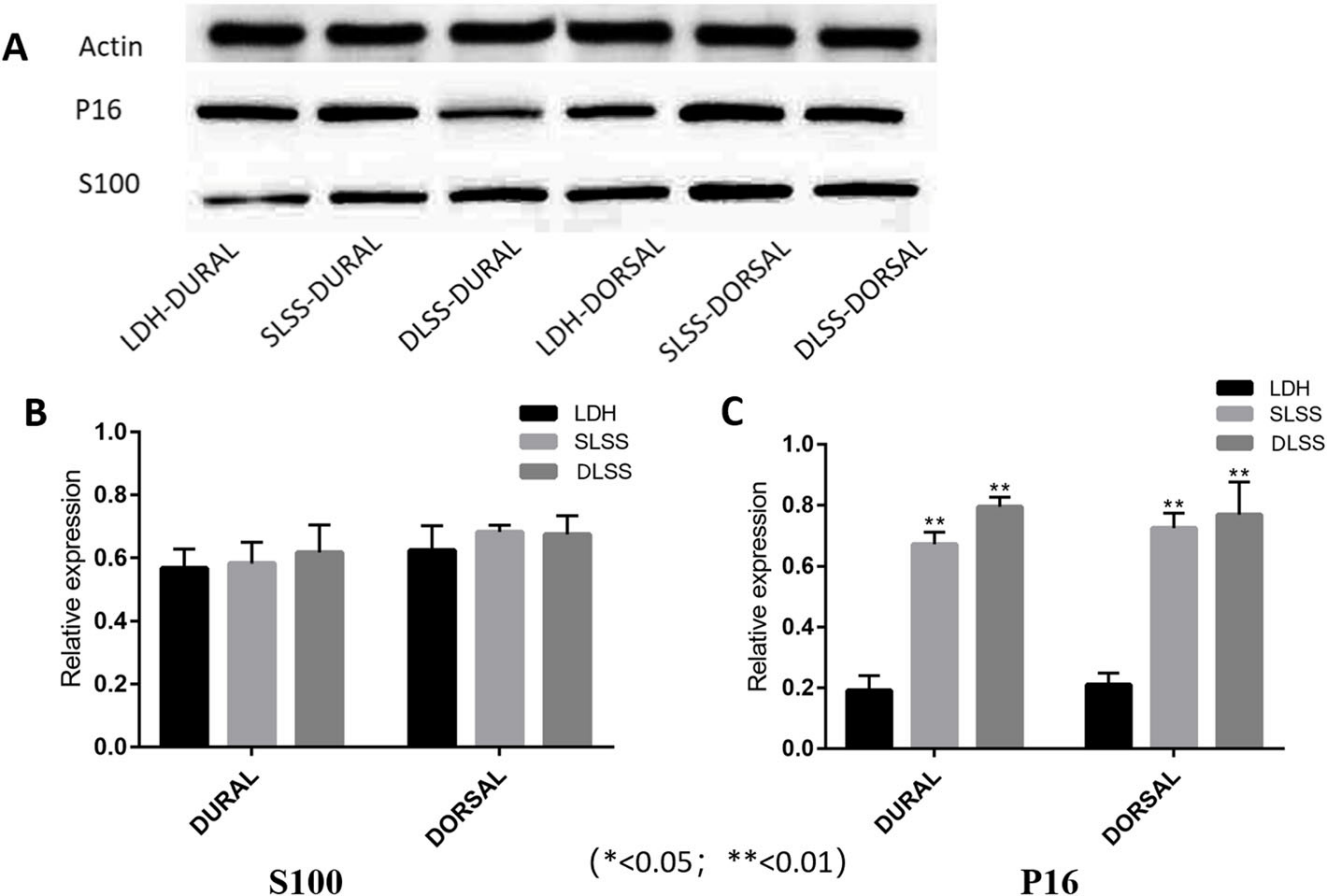
**a**: Detection of P16 protein and S100 protein expression by western blot. **b**: There was no significant difference in the expression of S100 mRNA in the three groups of LDH, SLSS and DLSS, and there was no significant difference in the dorsal or dura mater of LF cells in the three different groups. The mRNA expression of P16 in SLSS, DLSS group was significantly higher than that in LDH group(*p* < 0.01). The mRNA expression level of P16 in the dorsal ligament of DLSS group was much higher than that of SLSS group, showing significant difference(*p* < 0.05). There was no significant difference in the expression of P16 mRNA between the ventral ligament of SLSS group and that of DLSS group

Western-blot analysis was adopted to evaluate the differential expression of P16 and S100 proteins among all three groups. As displayed in Fig. 1c, the protein expressions of S100 in both the dorsal and dural side of the LF among all the three groups showed no significant differences, consistent with the mRNA expression. However, as shown in the Fig. 1d, the expression level of P16 protein in the LDH group was much lower than that in the SLSS and DLSS group (*p* < 0.01), on the dorsal side or the dural side. In both sides of the LF, the P16 protein expression in the DLSS group was higher than that in the SLSS group, regardless of there being no statistical significance between them. There was a significant correlation between absolute LF thickness, relative LF thickness and the protein expression of P16 (*r* = 0.671, *p* = 0.001 and *r* = 0.732, *p* = 0.000, respectively; Tables 4 and 5). No significant correlation was existed between absolute LF thickness, relative LF thickness and the protein expression of S100.

**Table 4:** Correlation between absolute LF thickness, relative LF thickness and the protein expression of P16 and S100

	P16		S100	
	*r*	*P*	*r*	*P*
Absolute LF thickness	0.671	0.001	0.396	0.084
Relative LF thickness	0.732	0.000	0.424	0.062

**Table 5:** Correlation between absolute LF thickness, relative LF thickness and the mRNA expression of P16 and S100

	P16		S100	
	*r*	*P*	*r*	*P*
Absolute LF thickness	0.633	0.003	−0.285	0.222
Relative LF thickness	0.590	0.006	−0.322	0.167

### The mRNA expression of P16 and S100 among LDH, SLSS and DLSS groups

**Fig. 2:**
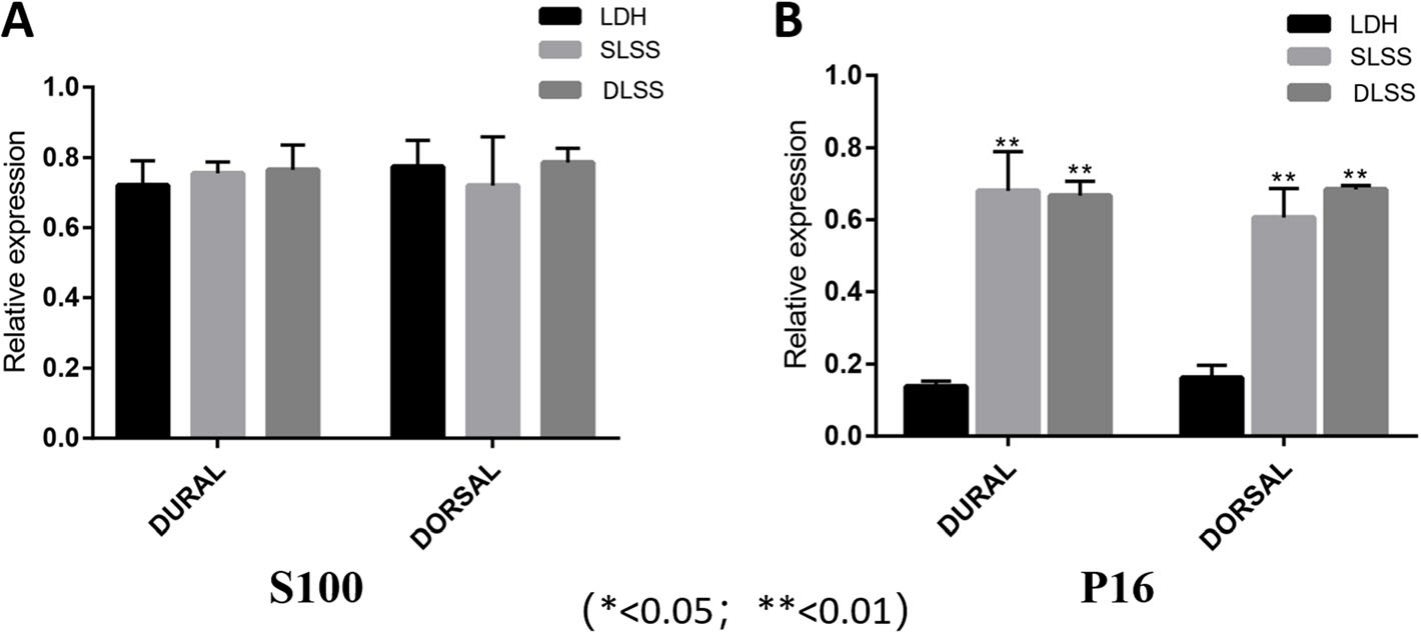
**a**: There was no significant difference in the protein expression of S100 in dorsal ligament and dura mater in LDH, SLSS and DLSS groups. **b**: The protein expression level of P16 in dorsal and dural side of LDH group was much lower than that of SLSS and DLSS group(*p* < 0.01). The protein expression of P16 in both sides of ligament in DLSS group was higher than that in SLSS group, although no statistical significance was noted

As illustrated in Fig. 2a, the mRNA expression of S100 obtained from the LF cells among the three different groups of LDH, SLSS and DLSS showed no significant differences, on the dorsal and dural side of the LF. However, the P16 mRNA expression in the group of SLSS was dramatically higher than that in the LDH (*p* < 0.01), and DLSS (*p* < 0.01) groups. Moreover, in the dorsal side of the LF, the expression level of P16 mRNA in the DLSS group was much higher than that in the SLSS group, and showed significant differences (*p* < 0.05, Fig. 2b) Conversely, in the dural side of the LF showed no significant differences between the SLSS and DLSS group. There was a significant correlation between absolute LF thickness, relative LF thickness and the protein expression of P16 (*r* = 0.633, *p* = 0.003 and *r* = 0.590, *p* = 0.006, respectively; Table 4 and 5). No significant correlation was noted between absolute LF thickness, relative LF thickness and the protein expression of S100.

## Discussion

MRI is an indispensable and important imaging method in the diagnosis and treatment of LSS. Safak et al. measured the thickness of LF at L4/5 level in 320 patients with LF hypertrophy, and the thickness of LF was between 1.8 and 5 mm [9]. Our results of LF thickness are higher than those of previous studies. This may be due to LF being subjected to more stress from changes in living habits in recent years and possibly related to race [10]. In the previous study, we measured the absolute and relative thickness of LF and found that the thickness of LF in DLSS group and SLSS group was significantly higher than that in LDH group [8]. In this study, we further investigated the correlation between the thickness of LF and the expression of P16 and S100. The correlation between the absolute thickness and relative thickness of LF and the expression of P16 protein and P16 mRNA was statistically significant. The correlation between S100 protein and S100 mRNA expression was not statistically significant. It is suggested that the expression of P16 may be related to the hypertrophy of LF.

Lonne and Cha found that P16 is a key factor in regulating the cell cycle from G1 to S phase [11]. P16 is rarely expressed in normal skin and mature scar [12]. However, in hyperplastic scar fibroblasts, P16 has a high level of expression [13]. In this study, the analysis of results showed that the expression of P16 protein in LDH group was significantly lower than that in SLSS group and DLSS group.. The expression of P16 protein in DLSS group was higher than that in SLSS group although not significantly. Combined with the results of this study, we speculate that the high expression of P16 may be related to the occurrence and development of LF hyperplasia.

Schräder et al. evaluated LF calcification and structural changes of elastic fibers in patients with LSS [14]. The results showed that LF showed significant calcification and fibrosis and decreased elastic/collagen fiber ratio. The pathological process of LF hypertrophy is similar to that of scar hyperplasia [15], which may be associated with local inflammatory response and fibroblast proliferation. The excessive proliferation of collagen fibers is related to the degradation of elastic fibers [16], which is consistent with the results of this experiment. Therefore, we consider that P16 is an important regulatory factor in the occurrence and development of LF hypertrophy and a key factor leading to LSS. The results of this study showed that there was no significant difference in the expression of P16 protein in dorsal LF between SLSS and DLSS groups, which was not consistent with that of previous studies. This may be due to the protein level not always being linear with its corresponding mRNA expression, which may be due to transcriptional and post-translational modification.

In our study, analysis showed that the mRNA expression of P16 in SLSS group and DLSS group was significantly higher than that in LDH group. On the dorsal side of LF, the mRNA expression of P16 in DLSS group was higher than that in SLSS group, but there was no significant difference in dural side of LF. It is suggested that the mRNA expression of P16 on the dorsal side is more sensitive than that on the dural side in reflecting the severity of LSS. Sairyo [17] found that the dorsal side of the LF is more likely to be enlarged than the dural side, which may be related to the greater stress and more active proliferation of the dorsal side. This is consistent with our results, and our study gives a further molecular explanation for this result. The mRNA expression of P16 was consistent with its protein expression and imaging findings in the three groups.

In previous studies, Zainuddin et al. found overexpression of P16 (INK4a) mRNA in human diploid fibroblasts [18]. Hypertrophic LF hyperplasia is caused by excessive proliferation of fibroblasts and imbalance of elastic/collagen fiber ratio [19]. Therefore, the high expression of P16 may be related to LF hypertrophy, which may indicates the LSS. This is consistent with the imaging results in our study. Yabe et al. studied the changes of elastic fibers and proteoglycans in LF [20]. It was found that the elastic fibers in the hypertrophic LF decreased and the proteoglycan increased. This pathological process mainly occurs in the dorsal side of LF, which is consistent with our study.

Yaundong et el., through in vitro studies, found that inhibiting the expression of S100A8 can inhibit the growth of fibroblasts in hypertrophic scars [7]. Zhao found that when S100A12 was inhibited by RNA interference keratinocytes, fibroblasts would not be activated [21]. It is suggested that S100A12 is a potential therapeutic target for skin fibrosis. Zhong found that S100A8/A9 was highly expressed in hypertrophic scar fibroblasts [22]. These findings suggest that S100 family proteins may be closely related to the activation of fibroblasts, resulting in LSS. However, in this study, there was no significant difference in S100 family mRNA and proteins between LDH, SLSS and DLSS groups, and between ventral and dorsal LF. this indicates the that the mechanism of LF hypertrophy is quite complex, and the role of S100 needs further research to confirm.

However, our research has some limitations, which include a small sample size taken from the yellow race in northern China. Expanding the scope of sample collection, increase contact and cooperation with national hospitals, and sample size can improve further studies. Furthermore, high expression of P16 after the development of LSS and LF hypertrophy prior to the surgical stage does not indicate that P16 runs through the entire LSS. In our future studies, samples from patients with non-operative stage of LF hypertrophy should be obtained to investigate the expression of P16 in patients with non-operative stage of LF hypertrophy.

## Conclusions

P16 is involved in the process of LF hypertrophy in patients with LSS. The expression of P16 may be related to the hypertrophy of LF, while the thickness of LF is related to the imaging expression of P16. No obvious evidence proves that S100 may be related to the hypertrophy of LF in patients with LSS.

## Supplementary information

**Additional file 1.**

**Additional file 2.**

**Additional file 3.**

## Data Availability

The current study used data collection and analysis can be reasonable request from the corresponding author.

## Abbreviations

LSS: Lumbar spinal stenosis
LF: Ligamentum flavum
LDH: Lumbar disc herniation
SLSS: Single-segment spinal stenosis
DLSS: Double-segment spinal stenosis
MRI: Magnetic Resonance Imaging
qPCR: Quantitative Polymerase Chain Reaction
