# Supplementary figures and images for: The correlation between imaging expression of P16 and S100 in hypertrophic ligamentum flavum
Source: BMC Musculoskelet Disord. 2020 Jun 8;21:359. doi: 10.1186/s12891-020-03395-y (PMC7282051; doi:10.1186/s12891-020-03395-y)

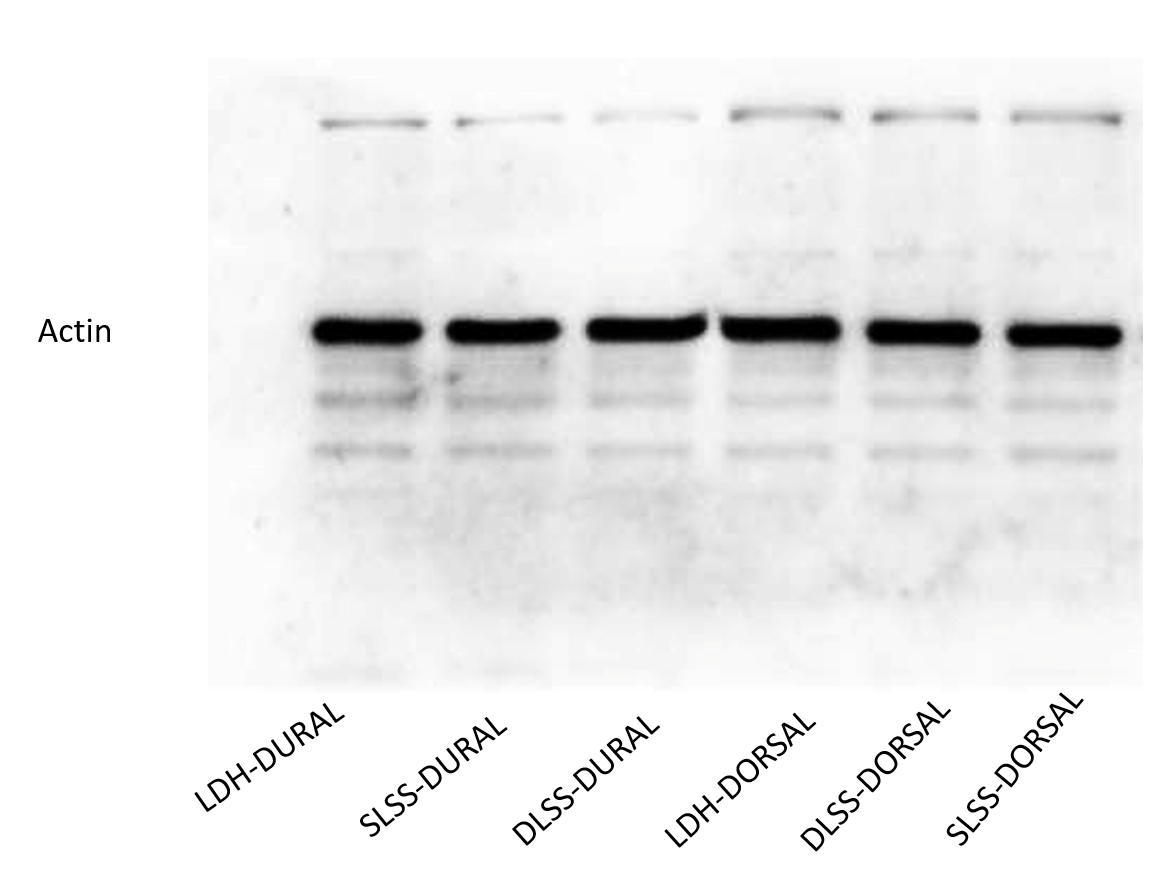

Supplement: Supplementary file 1 — Additional file 1. [file 12891_2020_3395_MOESM1_ESM.tif]

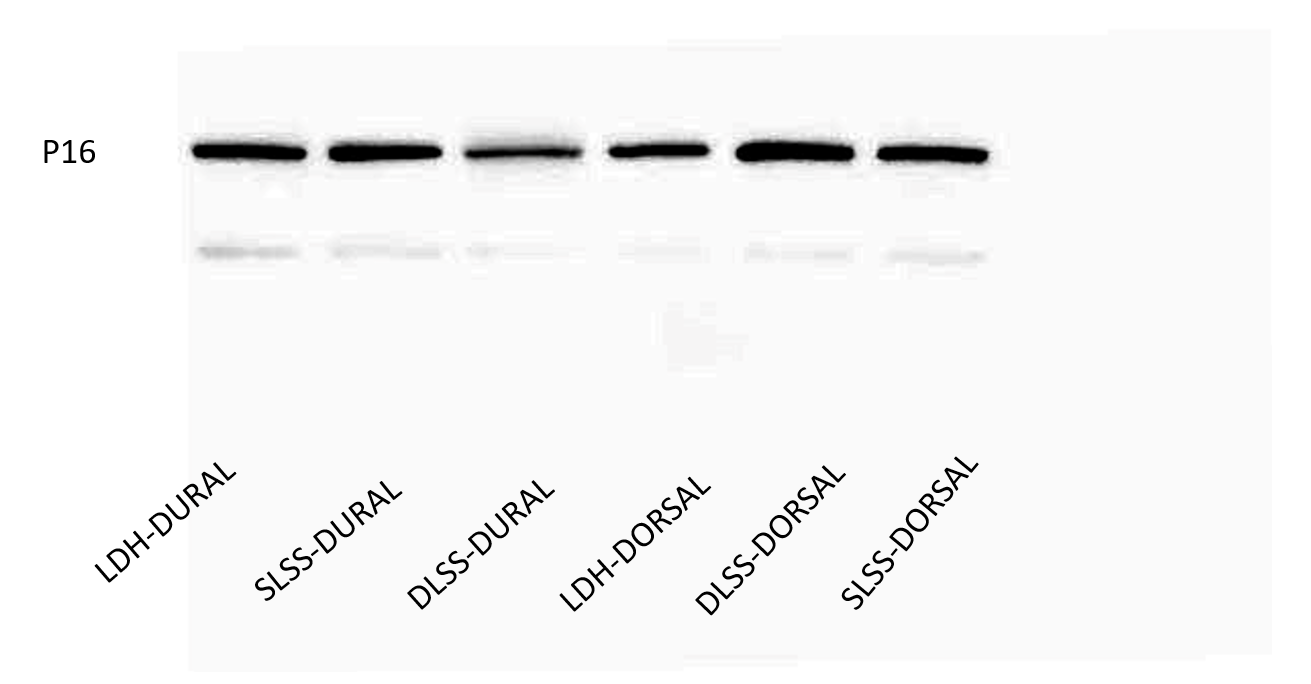

Supplement: Supplementary file 2 — Additional file 2. [file 12891_2020_3395_MOESM2_ESM.tif]

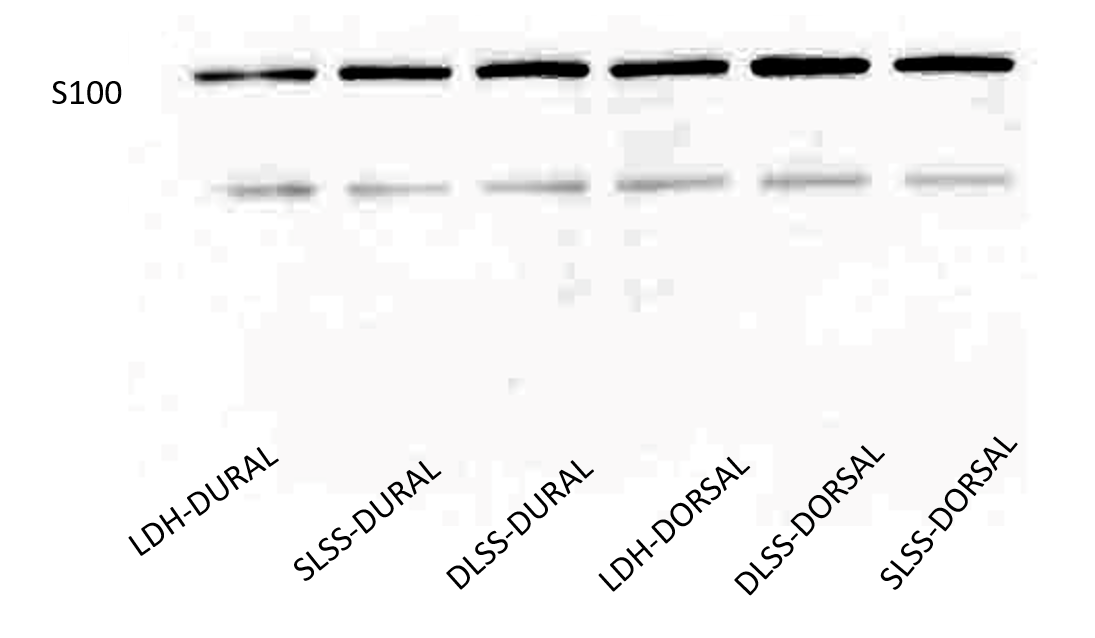

Supplement: Supplementary file 3 — Additional file 3. [file 12891_2020_3395_MOESM3_ESM.tif]
